# Photobiomodulation versus ischemic preconditioning for protection against exercise-induced muscle damage: protocol for a randomized controlled trial

**DOI:** 10.1007/s10103-026-05048-3

**Published:** 2026-09-26

**Authors:** Eduardo Lima, Rafael Pereira, Cláudia Thais Pereira Pinto, Felipe Sampaio-Jorge, Wouber Hérickson de Brito Vieira, Mikhail Santos Cerqueira

**Affiliations:** 1 Present Address: Southwest Bahia State University, Jequié, BA Brazil; 2 Present Address: Integrative Physiology Research Center, Southwest Bahia State University, Jequié, BA Brazil; 3 Researcher at the Laboratory of Research and Innovation in Sport Sciences Founder and Scientific Director of DoSys Academy, Universidade Federal do Rio de Janeiro, Rio de Janeiro, Brazil; 4 Present Address: Federal University of Rio Grande do Norte, Natal, Brazil; 5 Present Address: State University of Southwest Bahia, Postgraduate Program in Physical Education, Jequié, BA Brazil

**Keywords:** Eccentric exercise, Recovery, Muscle function, Randomized trial, Muscle soreness

## Abstract

Exercise-induced muscle damage (EIMD) occurs after unaccustomed eccentric exercise and may impair muscle recovery. Preconditioning with photobiomodulation (PBM) or ischemic preconditioning (IPC) may mitigate EIMD; however, no study has directly compared their effectiveness. This study aims to compare the effects of PBM and IPC applied prior to eccentric exercise on indirect markers of EIMD and associated perceptual responses. We hypothesized that MVIC trajectories would differ among the PBM, IPC, and sham groups, with PBM expected to provide greater preservation of MVIC than IPC. A prospective, randomized, placebo-controlled clinical trial with concealed allocation, blinded outcome assessors and statistician, and intention-to-treat analysis will be conducted. Healthy men (18–35 years) will be randomly allocated to PBM, IPC, or sham groups (n = 15 per group). After the intervention, participants will perform an eccentric exercise protocol for the elbow flexors consisting of 30 repetitions using a dumbbell load numerically matched to maximal voluntary isometric contraction (MVIC) values, with 45-second rest intervals. The primary outcome will be MVIC. Secondary outcomes include rate of force development, delayed-onset muscle soreness, pressure pain threshold, ultrasound echo intensity, range of motion, perceived exertion, and affective responses. Outcomes will be assessed at baseline, immediately after exercise (only MVIC), and at 24-, 48-, and 72-hours post-exercise. The primary analysis will assess the group × time interaction on MVIC using linear mixed-effects models, while secondary outcomes will be analyzed exploratorily. This study will provide, to our knowledge, the first direct comparison between PBM and IPC as preconditioning strategies to attenuate EIMD. The findings may help determine the most effective and clinically applicable intervention by considering both physiological and perceptual responses, thereby supporting decision-making in clinical and sports settings.

**Trial registration**

Brazilian Registry of Clinical Trials (REBEC), RBR-6gr5kf; registered February 19, 2020; amended August 26, 2026.

## Background

Exercise-induced muscle damage (EIMD) is a physiological response that commonly occurs after strenuous or unaccustomed eccentric exercise. It is characterized by structural and functional alterations in skeletal muscle and is typically reflected by indirect markers such as muscle strength, rate of force development, delayed-onset muscle soreness (DOMS), ultrasound echo intensity and range of motion [1–6]. Eccentric exercise involves muscle activation while the muscle is lengthened under load [7] and allows greater force production at a lower metabolic cost compared with concentric contractions [8–11]. Despite these advantages, eccentric contractions are strongly associated with the development of EIMD, particularly in individuals unaccustomed to this type of exercise. In sports settings, eccentric contractions are fundamental for actions such as sprinting, jumping, and rapid deceleration, whereas in rehabilitation programs they are frequently prescribed to improve muscle strength, tendon remodeling, and functional capacity. However, excessive EIMD may temporarily impair neuromuscular performance and delay the progression of training or therapeutic programs [12, 13].

Strategies to attenuate EIMD have therefore become an important focus in both athletic and clinical contexts. Post-exercise therapeutic approaches such as cryotherapy, massage, and compression garments are frequently used to accelerate recovery and help maintain training intensity and adherence to exercise programs [14–16]. More recently, strategies applied before exercise, known as preconditioning strategies, have been investigated as alternative approaches to mitigate the magnitude of EIMD [17–19]. Among these, photobiomodulation (PBM) and ischemic preconditioning (IPC) have received increasing attention due to their potential to modulate muscle metabolism, oxidative stress, and tissue perfusion, which are key mechanisms involved in the development of EIMD [20].

PBM using LASER (light amplification by stimulated emission of radiation) or LED (light emitting diode) devices employs non-ionizing light sources in the visible (red) or infrared spectrum to modulate biological processes [21]. Experimental and clinical studies suggest that PBM applied before exercise may improve muscle performance, reduce fatigue, and accelerate recovery from muscle damage [22, 23]. These effects are thought to be mediated by mechanisms such as increased mitochondrial activity and adenosine triphosphate (ATP) synthesis, improved microcirculation, and reduced oxidative stress [24]. In parallel, ischemic preconditioning (IPC) involves repeated cycles of vascular occlusion and reperfusion induced by cuff inflation and deflation, which trigger protective cellular responses [17, 25–27]. IPC has been widely investigated for its protective effects against ischemia-reperfusion injury and has more recently been explored as a strategy to improve exercise performance and recovery. The mechanisms proposed for IPC include enhanced mitochondrial efficiency, improved oxygen delivery, attenuation of ATP depletion, and metabolic adaptations associated with increased adenosine availability [27–29].

Despite growing interest in these interventions, the existing literature presents methodological inconsistencies that limit direct comparisons between studies. Previous investigations evaluating PBM or IPC in the context of EIMD have used heterogeneous populations, including trained athletes, recreationally active individuals, and clinical populations, as well as different exercise protocols and outcome measures. Furthermore, intervention parameters such as light wavelength and energy density in PBM, or total restriction pressure (TRP) and duration in IPC, vary substantially between studies [23, 30]. These methodological differences make it difficult to determine which intervention may be more effective for attenuating EIMD. Therefore, a direct comparison between PBM and IPC within a controlled experimental design is needed to clarify their relative effectiveness and support evidence-based decision-making in both clinical and sports settings.

In addition to physiological outcomes, practical and perceptual factors may influence the selection and applicability of preconditioning strategies. The use of PBM may be limited by the cost of equipment, availability of devices, and the need for trained personnel to administer the intervention [31]. In contrast, IPC is relatively accessible but requires longer application times (typically ≥ 40 min) and may induce discomfort due to the high TRP required [17]. These differences highlight the importance of examining not only indirect markers of EIMD (e.g., muscle function, DOMS, ultrasound echo intensity, and range of motion), but also perceptual responses such as discomfort, affective valence, and perceived effort, which may directly impact adherence and real-world applicability of these interventions [19, 32, 33]. Although recent studies have reported beneficial effects of PBM [23] and IPC [30] in reducing markers of EIMD, direct comparisons between these preconditioning strategies within the same experimental design are still lacking, highlighting the need for controlled trials addressing this question.

Therefore, the present study aimed to compare the effects of PBM and IPC as preconditioning strategies on indirect markers of EIMD. Specifically, the objectives were: (1) to compare the effects of preconditioning with PBM or IPC on muscle function, DOMS, ultrasound echo intensity, and range of motion after EIMD; and (2) to examine perceptual responses associated with the interventions, including discomfort and affective valence during preconditioning and perceived effort during the eccentric exercise protocol. Based on the proposed mechanisms and previous evidence, it was hypothesized that MVIC trajectories would differ among the PBM, IPC, and sham groups, with PBM expected to provide greater preservation of MVIC than IPC.

## Methods

### Study design

**Fig. 1:**
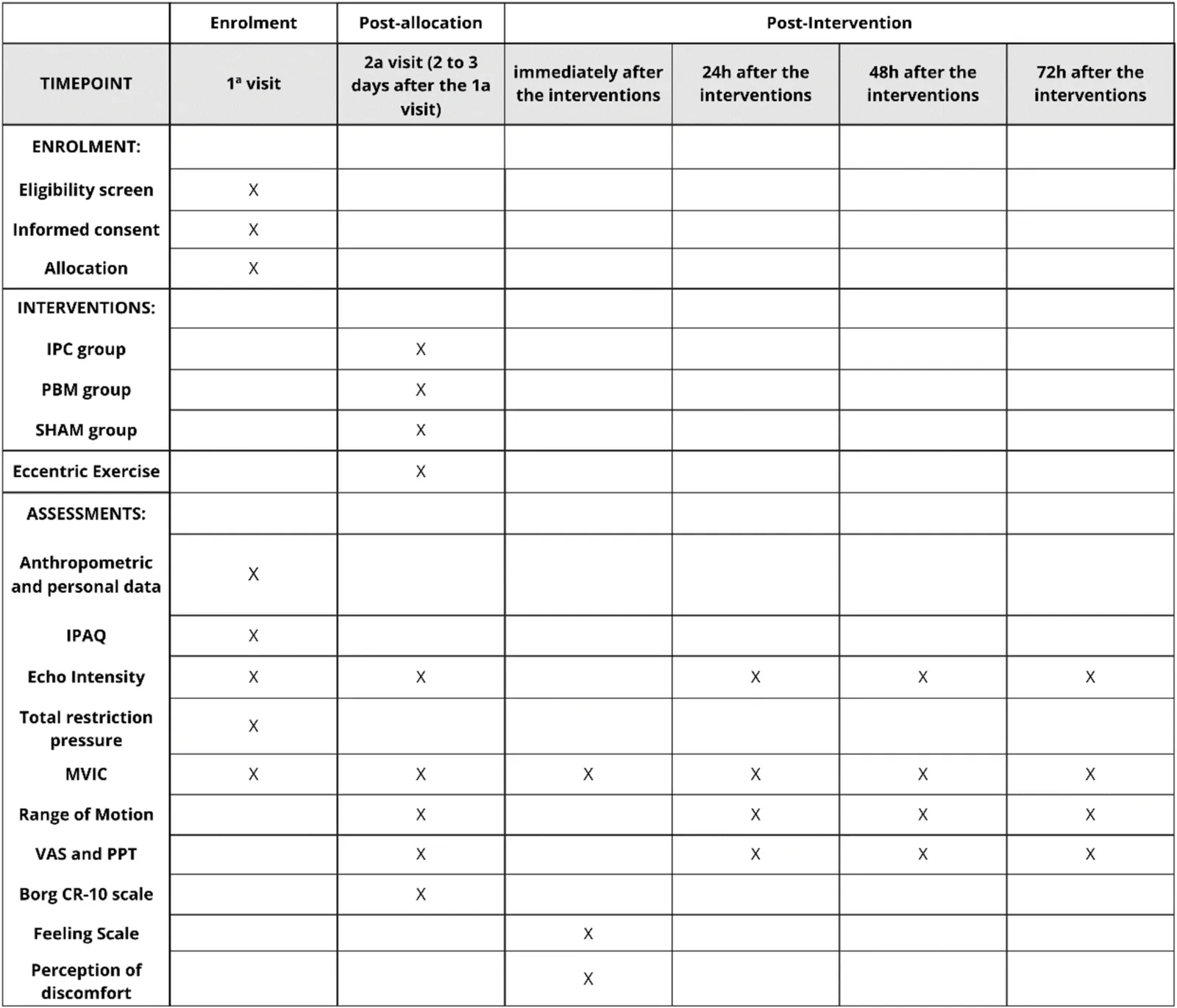
Study timeline according to the SPIRIT checklist recommendation. IPAQ: International Physical Activity Questionnaire; IPC: ischemic preconditioning; PBM: photobiomodulation; VAS: visual analogue scale; MVIC: maximal voluntary isometric contraction; PPT: pressure pain threshold

This will be a prospective, randomized placebo-controlled trial with concealed allocation. This protocol was developed in accordance with the Standard Protocol Items: Recommendations for Interventional Trials (SPIRIT) guidelines [34] (Fig. 1). In accordance with Resolution 466/12 of the National Health Council, the study protocol was initially approved by an institutional ethics committee in November 2019 and subsequently registered in the Brazilian Registry of Clinical Trials (REBEC) in 2020 (RBR-6gr5kf). However, the study was not initiated as originally planned due to the COVID-19 pandemic and a subsequent institutional change of the principal investigator. The protocol was therefore resubmitted and approved by the Research Ethics Committee of the State University of Southwest Bahia on December 13, 2022 (CAAE:65606522.2.0000.0055). During the subsequent methodological re-evaluation of the protocol, several aspects of the study design were refined, and an amendment to the REBEC registration was approved on August 26, 2026, updating the primary outcome, exercise model, target muscle group, intervention procedures, and outcome measures to reflect the current study design. All participants will provide written informed consent before participation.

At the time of submission, participant recruitment had not yet begun. Each participant will visit the university laboratory five times. During the first visit, personal and anthropometric data, including age, height, body mass, and BMI, will be collected. Moreover, data on physical activity levels, ultrasound imaging, blood pressure, total restriction pressure (TRP) and maximal voluntary isometric contraction (MVIC) will be measured. All procedures will be performed on the non-dominant upper limb. All preconditioning strategies will be applied with participants positioned in a supine position. All anatomical measurements involving the elbow flexor muscles (ultrasound imaging and pressure pain threshold) will be performed at the distal third of the distance between the lateral epicondyle and the acromion. Anatomical landmarks will be marked with a semi-permanent pen to ensure consistency across all assessments during the experimental period.

**Fig. 2:**
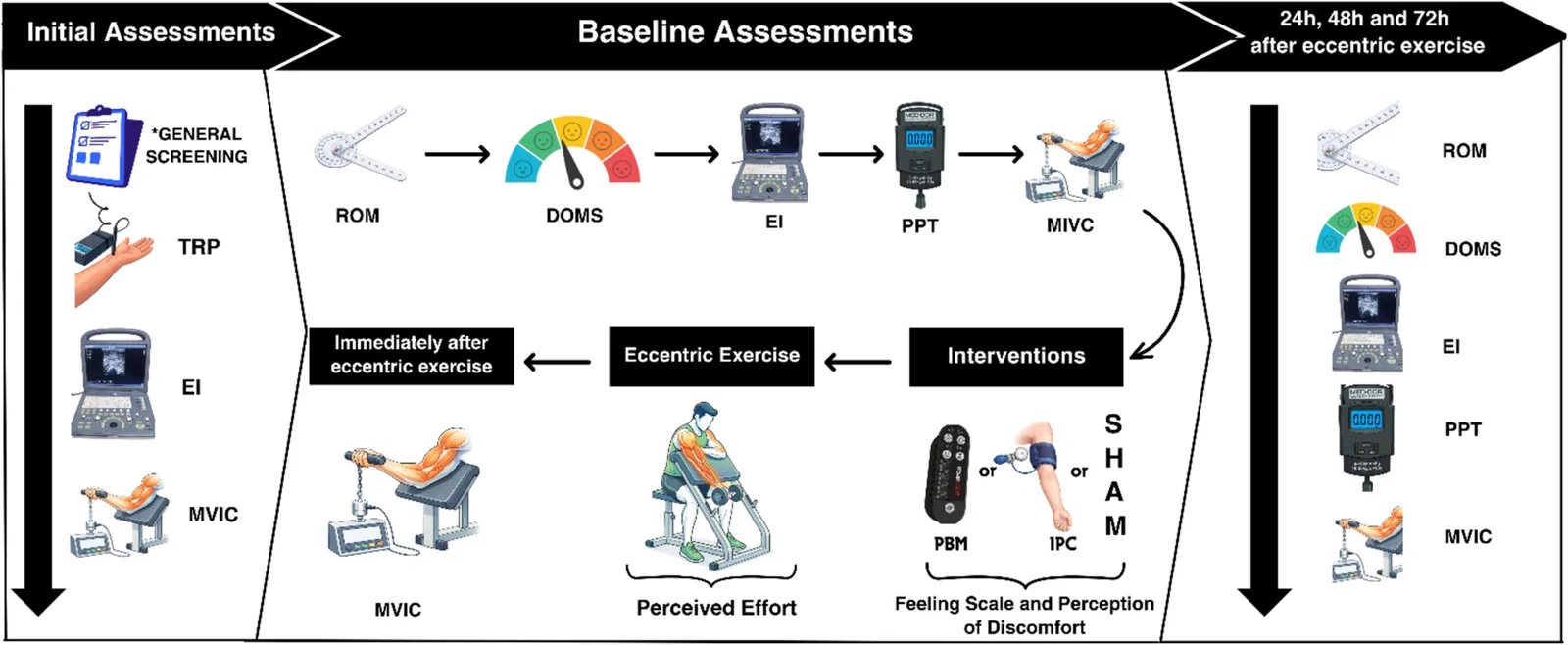
Experimental design. *General Screening: personal and anthropometric data, including age, height, body mass, data on physical activity levels, blood pressure. TRP: total restriction pressure; EI: echo intensity; MVIC: maximal voluntary isometric contraction. ROM: range of motion; DOMS: delayed-onset muscle soreness; PPT: pressure pain threshold; PBM: photobiomodulation; IPC: ischemic preconditioning

During the second visit, baseline outcome measures will be reassessed. After MVIC evaluation, the preconditioning strategies will be applied, followed by the eccentric exercise protocol. MVIC will be reassessed immediately after the eccentric exercise protocol. In the subsequent sessions (24, 48, and 72 h after the second visit), ultrasound imaging, DOMS, range of motion and MVIC will be evaluated. The experimental procedures and assessment timeline are summarized in Fig. 2.

Participant blinding will be attempted by informing individuals that the interventions may produce different sensory experiences, without providing detailed procedural information. To minimize bias, total intervention time and procedural sequence will be standardized across groups. All participants will undergo an IPC phase (40 min) followed by a PBM phase (8.4 min). The IPC group will receive active IPC and sham PBM; the PBM group will receive sham IPC and active PBM; and the sham group will receive sham IPC and sham PBM. Given that PBM typically produces minimal or no perceptible thermal or sensory effects, participants are unlikely to distinguish between active and sham conditions; blinding effectiveness will be assessed by asking participants to indicate their perceived group allocation (PBM, IPC, or sham). All assessments will be conducted at the same time of day for each participant to minimize the influence of circadian rhythms.

### Participants

Participants will be recruited through advertisements on social media. Interested individuals will complete an initial screening questionnaire to verify eligibility. Recruitment is planned to take place in Jequié, Bahia, Brazil. Inclusion criteria will be: healthy men aged 18–35 years, height between 1.65 and 1.85 m, body mass index (BMI) between 18.5 and 30 kg/m², and classified as irregularly active or active according to the short version of the International Physical Activity Questionnaire (IPAQ-SF) following standardized IPAQ scoring guidelines. Participants must also have no engagement in upper-limb strength training in the previous three months, no prior experience with ischemic IPC or blood flow restriction exercise, and be non-smokers. The height range was defined to minimize variability in limb length and potential mechanical leverage effects on MVIC measurements. Exclusion criteria will include a history of musculoskeletal injury in the upper limbs within 30 days prior to the experiments, regular use of vasoactive medications, and use of nutritional supplements that could influence vascular or neuromuscular responses.

Eligibility criteria will be verified during the initial screening questionnaire and confirmed during the first laboratory visit. Participants experiencing an unrelated musculoskeletal injury will discontinue the intervention as appropriate; however, they will remain eligible for the intention-to-treat analysis. Participants will be instructed during the first session to avoid strenuous exercise, alcohol consumption, and the use of pharmacological or therapeutic resources that could influence the outcomes for 48 h before each experimental session and throughout the experimental period. These instructions will be reinforced at the end of each visit.

Participants will be randomly assigned (1:1:1) to PBM, IPC, or sham groups using permuted-block randomization through web-based randomization software (Sealed Envelope Ltd., London, UK). Allocation will be concealed in sequentially numbered, sealed opaque envelopes prepared by an independent researcher before study initiation. Researchers responsible for determining TRP and administering the interventions will be unblinded because intervention delivery requires knowledge of group allocation, and will not participate in outcome assessments. Participant blinding to preconditioning allocation will be attempted. Outcome assessors and the statistician will remain blinded to group allocation and will not be present during the preconditioning procedures. Group codes will be revealed only after completion of the predefined analyses.

#### Risks, benefits, and adverse event monitoring

All participants will receive information on the benefits of physical exercise and may not experience any direct clinical benefit from participation. Muscle-performance assessments will be performed for research purposes. A team of physical therapists will be available to address any adverse events or discomfort related to the study procedures. Potential risks related to IPC include transient pain or discomfort, skin irritation or bruising, numbness or tingling, and, rarely, neurovascular symptoms associated with limb compression. Risks related to the eccentric exercise protocol include delayed-onset muscle soreness (DOMS), edema, and muscle fatigue. Only participants without contraindications to exercise or IPC will be included to mitigate these risks. Potential adverse events will be explained to participants by the researchers and detailed in the informed consent form. Participants will be encouraged to promptly report any occurrences or complaints related to the study procedures. Adverse events will be monitored during each experimental visit by the research team and recorded in standardized forms. The IPC intervention will be immediately discontinued if participants report intolerable pain, persistent numbness or tingling, weakness, or other symptoms suggestive of neurovascular compromise. If a significant adverse event occurs, the intervention will be interrupted and appropriate medical evaluation will be provided. Serious adverse events requiring medical evaluation or hospitalization will be documented and reported.

### Primary outcome

#### Maximal voluntary isometric contraction

Isometric peak force, expressed in kilogram-force (kgf), will be obtained during the MVIC maneuver. The MVIC of the elbow flexor muscles will be preceded by unilateral warm-up with submaximal isometric contractions (three repetitions lasting six seconds each) in all evaluation days. After warming up, each volunteer will be instructed to sit on a custom-made barbell curl bench with the shoulder joint angle at 45° of flexion and 0° of abduction. After positioning, the volunteers will pull a handle attached to a load cell (EMG System, São José dos Campos, São Paulo, Brazil). The elbow joint angle will be fixed at 90°, and the participant will be asked to flex the elbow joint as much as possible, keeping the forearm supinated. Participants will be instructed to flex the elbow as quickly and forcefully as possible upon hearing the verbal command “go”, sustain the contraction for five seconds, and then relax upon the command “stop”. A strong verbal command will be offered by the researchers [35]. This measurement will be performed three times before the pre-conditioning interventions, with a 45-s interval between attempts. The highest MVIC obtained during the baseline assessment will be considered the reference value. MVIC will be recorded in its original measurement scale for the primary analysis. For exploratory interpretation, post-exercise MVIC values will also be expressed as percentage change from baseline.

### Secondary outcomes

#### Rate of force development

Rate of force development will be analyzed to provide insights into neural contributions (0 to 50 ms) and muscular contributions (100 to 200 ms) to strength generation and neuromuscular fatigue. Rate of force development will be calculated from the force–time curve obtained during MVIC using previously described methods [35–37]. Data processing and analysis will be performed using MATLAB software (MathWorks, Natick, MA).

#### Ultrasound echo intensity

Ultrasound imaging will be performed using a Figlabs FP 102 device, maintaining the same positioning used during TRP determination. With the tested arm relaxed, a linear ultrasound transducer (7.0 MHz, 75 dB gain) coated with acoustic gel will be positioned perpendicular to the longitudinal axis of the biceps brachii at the standardized anatomical location described above. Three consecutive images will be obtained at this site by the same experienced researcher and averaged for analysis. Echo intensity will be quantified from ultrasound images using the grayscale histogram function in ImageJ software (National Institutes of Health, USA) [38].

#### Delayed-onset muscle soreness and pressure pain threshold

DOMS of the elbow flexor muscles will be assessed using a visual analogue scale ranging from 0 mm (“no pain”) to 100 mm (“extreme pain”). DOMS will be evaluated during maximal passive elbow extension, at which point participants will indicate their perceived pain level on the visual analogue scale [39, 40]. A new visual analogue scale sheet will be provided for each assessment to prevent participants from referencing previous scores.

Pressure pain threshold will be measured using a pressure algometer (MedDor, Minas Gerais, Brazil). Participants will be positioned supine on an examination table, with the tested arm relaxed alongside the body, the elbow fully extended, and the forearm in a supinated position. The arm will be gently stabilized by the examiner to minimize unwanted movement. Pressure will be applied perpendicularly to the biceps brachii (distal third of the distance between the lateral epicondyle and the acromion), increasing at a rate of 1 kg/s until the participant reports the onset of pain, which will be considered the mechanical pain threshold. The measurement will be repeated three times at the same location, and the average value will be used for analysis [41]. A 30-second rest interval will be allowed between consecutive measurements.

#### Range of motion

Elbow range of motion will be assessed with participants standing, the arm relaxed alongside the body, and the forearm in a supinated position. The angle will be determined by calculating the angular difference between the relaxed arm position and maximal active elbow flexion. Measurements will be obtained using a plastic goniometer (Carci, SP, Brazil), with the axis aligned with the lateral epicondyle of the humerus and the goniometer arms positioned parallel to the humerus and forearm, extending toward the radial styloid process [42].

#### Perceived discomfort and affective valence

The subjective perception of discomfort will be measured using a visual analogue scale, as previously described. Affective valence (pleasure/displeasure) related to the IPC, PBM, or sham interventions will be assessed using the Feeling Scale, an 11-point scale ranging from − 5 (very poor) to + 5 (very good) [15]. Both scales will be applied immediately after the preconditioning strategies.

#### Perceived effort

The subjective perception of effort will be measured by the modified Borg Category-Ratio Scale (Borg CR-10 scale) [43]. This scale ranges from 0 (“rest state”) to 10 (“maximum effort”). Participants will use this scale to quantify the effort required to complete the eccentric exercise protocol, and it will be administered to all participants immediately after the eccentric exercise protocol [43, 44]. Participants will be instructed to focus on the overall effort exerted to complete the exercise protocol.

### Interventions

#### Ischemic preconditioning

The TRP will be determined at rest using a portable Doppler ultrasound device (DV 6010B Medmega, Franca-SP). Participants will be instructed to avoid strenuous exercise, caffeine, and alcohol for 24 h before the assessment. For TRP determination, participants will rest in a quiet, temperature-controlled room (23 °C) for 10 min prior to the procedure. During the evaluation, participants will remain supine with both upper and lower limbs resting on the exam table. The Doppler transducer will be positioned on the anterior aspect of the wrist, medial to the distal end of the radius, over the anatomical course of the radial artery. A BFR nylon cuff (Arm Cuffs V2.1, Fit Cuffs^®^, Denmark; cuff width 7 cm) will be placed near the axillary region and inflated based on a prior protocol [45]. TRP will be defined as the pressure at the time when arterial pulse is abolished, which will be indicated by the absence of auscultatory signal.

**Fig. 3:**
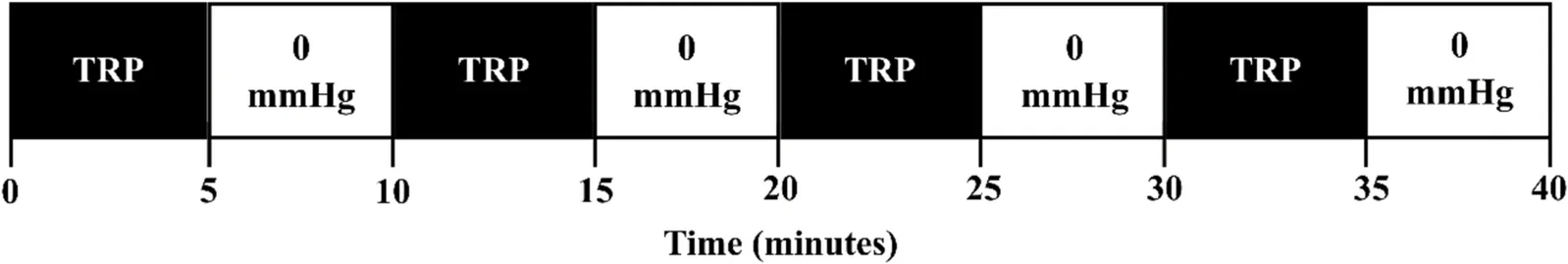
Ischemic preconditioning protocol

IPC will be performed immediately after the pre-exercise MVIC test. The IPC procedure will last 40 min (Fig. 3) and will consist of four cycles of 5 min of TRP, each followed by 5 min of reperfusion (0 mmHg) [46]. The procedure will be performed using the same cuff and participant positioning described for the determination of TRP [47]. This IPC protocol has previously been proposed as having a potential to attenuate exercise-induced muscle damage [48]. Following the completion of the IPC procedure, a sham application of PBM will be performed. After this procedure, participants will proceed to the eccentric exercise protocol.

#### Photobiomodulation preconditioning

PBM will be delivered using the Deep Light LED Device (Avanutri^®^, Três Rios, RJ, Brazil). The LED cluster, measuring approximately 12.2 cm in length, will be positioned in direct contact with the skin over the biceps brachii muscle at a 90° angle, with light pressure applied. The cluster will be positioned to cover the distal third of the distance between the lateral epicondyle and the acromion. Detailed PBM parameters are presented in Table 1. An energy of 72 J per diode (576 J total for the eight-diode cluster) was selected based on previous evidence that higher doses of infrared LED irradiation may attenuate the decline in MVIC [49].

**Table 1:** Parameters of preconditioning with LED photobiomodulation

Device characteristics	
LED wavelength	810 nm (infrared)
Emission mode	Continuous wave
Number of diodes	1 cluster (8 diodes)
Distance between diode centers	2.0 cm
Beam divergence angle	15°
Geometric parameters	
Spot diameter (at emitter)	6.30 mm
Spot area (at skin surface)	0.71 cm² (estimated from beam divergence angle)
Total irradiation area (cluster aperture)	31.50 cm²
Power and irradiance	
Power per diode	150 mW (± 10%)
Total output power	1143 mW (measured output)
Peak irradiance (per diode at emitter surface)	201.30 mW/cm²
Average irradiance (cluster level)	36.30 mW/cm²
Dosimetry	
Application points	Single
Energy per diode	72 J
Irradiation time	504 s
Total energy (8 diodes)	576 J
Fluence at skin (per diode)	101.41 J/cm²
Average fluence (cluster level)	18.29 J/cm²

*Abbreviations*: *nm *nanometers, *cm *centimeters, *mm *millimeters, *mW *milliwatts, *J *joules, *s *seconds

#### Sham preconditioning

The sham applications will simulate IPC and PBM procedures without producing physiological effects. For the PBM sham condition, participants will wear protective eyewear and headphones emitting white noise to eliminate potential visual and auditory cues associated with device operation. The PBM device will be positioned and operated following the same procedures used in the active PBM condition; however, the LED emission will be disabled so that no therapeutic light is delivered while maintaining identical device handling and application time.

For the IPC sham condition, the cuff will be inflated to 10% TRP. This pressure is considered insufficient to meaningfully restrict arterial or venous blood flow and has been widely used in previous studies as a sham condition for ischemic preconditioning strategies [27]. Standardized instructions will be provided to all participants to maintain consistency between active and sham conditions.

### Eccentric exercise protocol

Muscle damage will be induced using a protocol consisting of 30 eccentric actions of the elbow flexors with dumbbells, based on a previous study [50]. The dumbbell load will correspond directly to the highest MVIC value obtained during baseline testing. For example, a participant presenting an MVIC of 10 kgf will perform the protocol using a 10-kg dumbbell. This approach has previously been shown to induce significant EIMD while remaining safe for participants [50]. A 45-s rest interval between repetitions will be adopted to allow partial recovery while maintaining sufficient mechanical stress to induce muscle damage. During each repetition, participants will lower the dumbbell from approximately 50° of elbow flexion to 170° of extension over 4–5 s. After each contraction, the investigator will remove the dumbbell and assist the participant in returning the arm to the starting position, ensuring that the movement remains exclusively eccentric. The interval between the preconditioning strategies and the eccentric exercise protocol will be approximately 5–15 min. The eccentric exercise protocol is expected to last approximately 25–30 min, including rest intervals. The main experimental visit, including baseline assessments, preconditioning procedures, eccentric exercise, and post-exercise assessments, is expected to last approximately 2 h.

### Statistical analysis plan

Sample size was determined a priori using data from an independent pilot study that applied the same eccentric exercise protocol as the present trial. The pilot included 20 participants (five per group) allocated to placebo, 6 J, 24 J, or 72 J PBM, with MVIC assessed before and immediately, 24, and 48 h after exercise. The pilot MVIC data yielded a within-participant correlation of 0.79, a Greenhouse–Geisser correction of ε = 0.82, and an observed group × time effect of f ≈ 0.23. The pilot-derived effect size was estimated from the group × time interaction using a repeated-measures ANOVA framework. Given the small pilot sample and the absence of an IPC group and 72-hour assessment, a conservative planning effect size of f = 0.12 was adopted. Using G*Power 3.1 (Heinrich-Heine-Universität Düsseldorf, Germany), the sample size was calculated for a three-group repeated-measures design with five measurements, α = 0.05, 80% power, f = 0.12, a within-participant correlation of 0.79, and ε = 0.82. The resulting minimum sample was approximately 44 participants; therefore, 45 participants (15 per group) were planned to ensure balanced allocation. Although the calculation used a repeated-measures ANOVA framework, linear mixed-effects models were prespecified for the primary analysis because they better accommodate within-participant correlations and incomplete repeated measurements.

Normality and homoscedasticity of residuals will be evaluated through graphical inspection (Q-Q plots and residual-versus-fitted plots) and the Shapiro–Wilk test. Potential influential observations and model fit will also be examined using standard diagnostic procedures. Descriptive statistics will be presented as mean ± standard deviation and 95% confidence intervals. The primary endpoint will be MVIC. The primary hypothesis concerns differences in the post-exercise MVIC recovery trajectory among the PBM, IPC, and sham groups. The primary inferential test will be the group × time interaction from a linear mixed-effects model including group and time (treated as categorical factors) as fixed effects, with a participant-specific random intercept to account for within-participant correlations. The prespecified key contrast will be the between-group difference in change in MVIC from baseline to 72 h between the PBM and IPC groups. Additional pairwise contrasts (PBM vs. sham and IPC vs. sham) and analyses of secondary outcomes will be considered exploratory and will not be subject to multiplicity adjustment. The effect measure for the PBM versus IPC comparison will be the between-group difference in change in MVIC from baseline to 72 h, reported with its 95% confidence interval and standardized effect size. Secondary continuous outcomes will be analyzed using linear mixed-effects models and interpreted as exploratory. For ordinal or bounded outcomes, generalized linear mixed-effects models with an appropriate distribution and link function will be used when the assumptions of a Gaussian model are not satisfied.

All randomized participants will constitute the intention-to-treat population and will be analyzed according to their originally assigned groups, regardless of intervention adherence or outcome availability. Missing observations will be handled within the prespecified mixed-effects framework using maximum likelihood estimation under a missing-at-random assumption. Sensitivity analyses comparing the primary maximum-likelihood analysis with a complete-case analysis will be performed if missing outcome data exceed 10% of the primary outcome observations. Statistical significance will be set at *p* < 0.05 for all analyses.

## Discussion

This study protocol is designed to compare the effectiveness of PBM and IPC as preconditioning strategies to attenuate EIMD. It is hypothesized that PBM may provide greater attenuation of EIMD than IPC, as well as more favorable perceptual responses. Although previous studies have reported beneficial effects of both PBM and IPC on markers of EIMD [23, 30], findings remain heterogeneous and direct comparisons between these interventions are lacking. Therefore, the present trial is expected to help clarify their relative effectiveness and address an important gap in the literature through a controlled head-to-head comparison within the same experimental design. If both interventions are found to be equally effective, PBM may offer greater practical advantages in terms of feasibility. For example, in the present protocol, PBM will require 8.4 min of irradiation, whereas IPC will require 40 min and may generate discomfort due to the applied occlusion pressure.

In addition to physiological outcomes, this study will also examine perceptual responses, which may play a relevant role in the applicability of preconditioning strategies in real-world settings. Discomfort, affective valence, and perceived effort may influence adherence and acceptance of these interventions in both clinical and sports contexts. Therefore, the inclusion of these outcomes is expected to provide a more comprehensive understanding of the potential benefits and limitations of PBM and IPC beyond traditional markers of muscle damage.

This study protocol was developed following rigorous methodological standards, including allocation concealment, blinded participants, outcome assessment and statistical analysis, and intention-to-treat analysis. Furthermore, the use of individualized TRP is also expected to improve internal validity by standardizing the stimulus across participants.

This study has some limitations. First, the inclusion of only male participants may limit the generalizability of the findings to other populations, such as women and older adults. However, this approach was adopted to minimize potential hormonal influences on muscle strength and pain perception. Additionally, the restriction to a narrow height range, although potentially limiting external validity, was implemented to reduce variability in upper-limb lever arms and improve the consistency of MVIC measurements. Second, the study will assess only the relative percentage of TRP rather than the actual percentage of blood flow occlusion, which prevents precise quantification of the degree of vascular restriction. Another limitation is the absence of circulating biomarkers, such as creatine kinase. Nevertheless, EIMD will be evaluated using several well-established indirect markers, including MVIC, DOMS, ultrasound echo intensity, and range of motion. Finally, sensory differences between IPC and sham IPC may allow some participants to infer their group allocation. However, sham procedures were designed to mimic the active interventions, and blinding effectiveness will be assessed at the end of the study.

Overall, this study is expected to provide relevant evidence regarding the comparative effectiveness and practical applicability of PBM and IPC as preconditioning strategies. The findings may contribute to improving decision-making in both clinical and sports settings, particularly in the selection of feasible and well-tolerated interventions to mitigate EIMD.

## Conclusion

This study protocol describes a randomized controlled trial designed to compare photobiomodulation and ischemic preconditioning as preconditioning strategies to attenuate exercise-induced muscle damage. The results of this trial are expected to clarify their relative effectiveness and provide clinically relevant information regarding both physiological and perceptual responses. These findings may support evidence-based decisions in rehabilitation and sports practice. 

## Data Availability

No datasets were generated or analysed during the current study.
